# Acute, dose–response effects of guayusa leaf extract on mood, cognitive and motor-cognitive performance, and blood pressure, heart rate, and ventricular repolarization

**DOI:** 10.1080/15502783.2024.2379424

**Published:** 2024-07-16

**Authors:** Nathaniel J. Helwig, Laura E. Schwager, Alexander C. Berry, Anna C. Zucker, Jacob S. Venenga, Samantha C. Sterbenz, Nathaniel D.M. Jenkins

**Affiliations:** a University of Iowa, Department of Health and Human Physiology, Iowa City, IA, USA; b University of Iowa, Abboud Cardiovascular Research Center, Iowa City, IA, USA

**Keywords:** Caffeine, dietary supplementation, cognition, cardiovascular, tea extract

## Abstract

An emerging sector of the nootropic market is tea-based nutraceutical products, but there are limited data assessing the dose-dependent effects of these products.

**Purpose:**

We conducted a randomized, double-blind, placebo-controlled crossover trial in young adults to examine the dose-dependent (600 mg versus 1200 mg), acute effects of consumption of an *Ilex guayusa* tea extract (GLE) on mood, cognitive and motor-cognitive performance, as well as its acute cardiovascular effects.

**Methods:**

Twenty-five adults (mean ± SD, age = 28 ± 7 y; 9 M/16 F) completed familiarization and then three randomly ordered experimental visits where they consumed either 600 mg (GLE_600_) or 1200 mg (GLE_1200_) GLE or placebo (PLA). Following supplement consumption, participants completed a mood state survey, assessments of perceived jitteriness, energy, and focus, and neurocognitive and motor-cognitive testing. Blood pressure (BP), heart rate, and QT interval length were determined before and after supplementation.

**Results:**

GLE_600_ significantly improved total mood disturbance (mean ± SE difference = -6.9 ± 2.6 au, *p* = 0.034), fatigue-inertia (−2.84 ± 0.89 au, *p* = 0.008), perceived energy (+13.00 ± 4.49 au; *p* = 0.02), motor speed (+4.52 ± 1.42 au, *p* = 0.008), and psychomotor speed (+7.20 ± 2.16 au, *p* = 0.005) relative to PLA. GLE_1200_ also improved psychomotor speed (+5.08 ± 2.16 ms, *p* = 0.045) and uniquely increased motor-cognitive performance as reflected by a decrease in reaction time (−0.106 ± 0.04 ms, *p* = 0.026) during a neurocognitive hop test. The effect of GLE on jitteriness was both dose- and sex-dependent. Jitteriness increased with increasing GLE dose in women only (*p* < 0.001). Both GLE_600_ and GLE_1200_ similarly increased systolic and diastolic BP by 4–5 mmHg (*p* ≤ 0.022). Neither GLE_600_ nor GLE_1200_ acutely influenced QTc length (*p* = 0.31).

**Conclusions:**

The goal of GLE supplementation should be considered when selecting a dosing strategy. Lower dosages of GLE (e.g. 600 mg) appear to optimize cognitive and mood-related outcomes while limiting side-effects such as jitteriness in women, and higher dosages may be necessary (e.g. 1200 mg) to promote improvements in motor-cognitive performance.

## 1. Introduction

Products marketed to enhance energy, focus, and mood have been on the market for decades, but the number of options has increased dramatically over the last 20 years alongside substantial increases in consumption, particularly among young adults. For example, consumption of caffeine-based energy drinks increased 11-fold among young adults from 2003 to 2016 [1], and the energy drink market is now evaluated at just over $86.35 billion with an expected growth rate of 8.3% annually until the year 2030 [2]. These dramatic increases in consumption likely coincide with the increasing cultural valuation placed on productivity and work performance and the reported ability of caffeinated products to improve cognitive performance and mood [3–6].

Nootropics are reported to support mood [7] and improve cognitive performance by augmenting cognitive function, cognitive flexibility, concentration, and attention [8,9] and are frequently incorporated into energy drink formulations. An emerging sector of the nootropic market is tea-based energy drinks. This advent is likely a result of their unique, naturally-occurring components with demonstrated pharmacological effects, [10] such as biologically active polyphenols that include chlorogenic acids (CGA), the ester of caffeic and qunic acid that is naturally found in high abundance in coffee and certain tea leaves [11,12]. While *Ilex guayusa* teas naturally contain 3–5% CGA, [10] novel guayusa leaf extracts (GLE) have been uniquely extracted to standardize for and increase the CGA content (e.g. 30% CGA) to maximize CGA-related benefits. In addition to its antioxidant effects, [10] CGA derivatives have been shown to bind to adenosine transporters and also to inhibit adenosine reuptake at a potency that is greater than the antagonistic binding affinity of caffeine with the adenosine A_2A_ receptor. [13] It has also been reported that even when matched for caffeine content and producing equivalent increases in serum caffeine concentrations, GLE produces a smaller increase in urinary epinephrine than synthetic caffeine. [14] Thus, it has been proposed that caffeine-containing sources that are high in CGA content (e.g. coffee, GLE standardized for CGA) may promote unique effects, perhaps promoting similar mood and performance-based nootropic benefits while limiting negative caffeine-induced side effects such as anxiety and jitteriness [11,12,15].

The primary mechanism for caffeine’s ergogenic effects is via antagonism of adenosine receptors, which indirectly alters the release of neurotransmitters that include dopamine, serotonin, and norepinephrine, heightening arousal [16]. Improvements in mood have been shown when caffeine is consumed at moderate dosages (2–5 mg/kg), whereas studies assessing higher doses of caffeine (5–8 mg/kg) report increases in tension and anxiety [8]. Recent evidence also suggests that chlorogenic acids alone may augment mood and behavior [12]. Bloomer et al. [17] observed that acute GLE supplementation did not significantly augment mood when compared to placebo or synthetic caffeine consumption. However, this study implemented a single, relatively high GLE dose (1080 mg, 270 mg caffeine) and given that the matched dose of synthetic caffeine in that study also did not produce significant effects, the effects of GLE on cognition and mood remain unclear.

In addition to potential effects on cognitive function and mood, GLE may also support other cognitive processes including complex movement and psychomotor skills. As these processes are heavily involved in physical function, it is paramount to investigate the potential link between the perceived benefits of GLE consumption and physical function [18,19]. Crucially, motor-cognitive (or neurocognitive) testing has been proven to be a reliable way to evaluate the interaction between physical and mental performance, as it challenges an individual’s ability to multitask and emphasizes visual-spatial processing during the performance of simple and complex motor tasks [18]. No prior studies have examined the effect of GLE on motor-cognitive performance.

It is also important to understand the potential adverse effects of GLE, especially regarding cardiovascular safety. Of the constituents, caffeine has the highest potential to cause adverse effects by altering hemodynamic and electrocardiographic parameters [5,20–22]. While Bloomer et al. [17] reported that a 1,080 mg dose of GLE elicited modest 6–9 mmHg increases in systolic blood pressure during video game play that were comparable to synthetic caffeine, it is not currently clear how GLE influences electrocardiographic parameters. Notably, the corrected Q-wave to T-wave interval (QTc) is an indirect measure of ventricular repolarization and indicator of cardiac arrhythmia risk [23,24], and energy drink consumption has been shown to acutely increase the QTc by ~8 ms relative to placebo which has been hypothesized to explain the previous links between energy drink consumption and sudden cardiovascular events [20]. It also appears that the potential for energy products to produce worrisome electrocardiographic changes is dependent on the dose consumed [20,25]. Thus, it is important to examine the acute cardiovascular effects of GLE consumption, and evaluation of the dose-dependent effects is particularly important.

We conducted a randomized, double-blind, placebo-controlled crossover trial in young adults to examine the dose-dependent effects of acute GLE consumption on cognitive and motor-cognitive performance, and mood, as well as its acute cardiovascular effects. We hypothesized that GLE would improve cognitive and motor-cognitive performance, and positive mood states such as vigor-activity in a dose-dependent manner without affecting negative mood states such as tension-anxiety. We also hypothesized that GLE would increase blood pressure in a direct, dose-dependent manner, but that there would be no effect on heart rate or the QTc [5,26].

## 2. Methods

### 2.1. Participants

**Figure 1.**
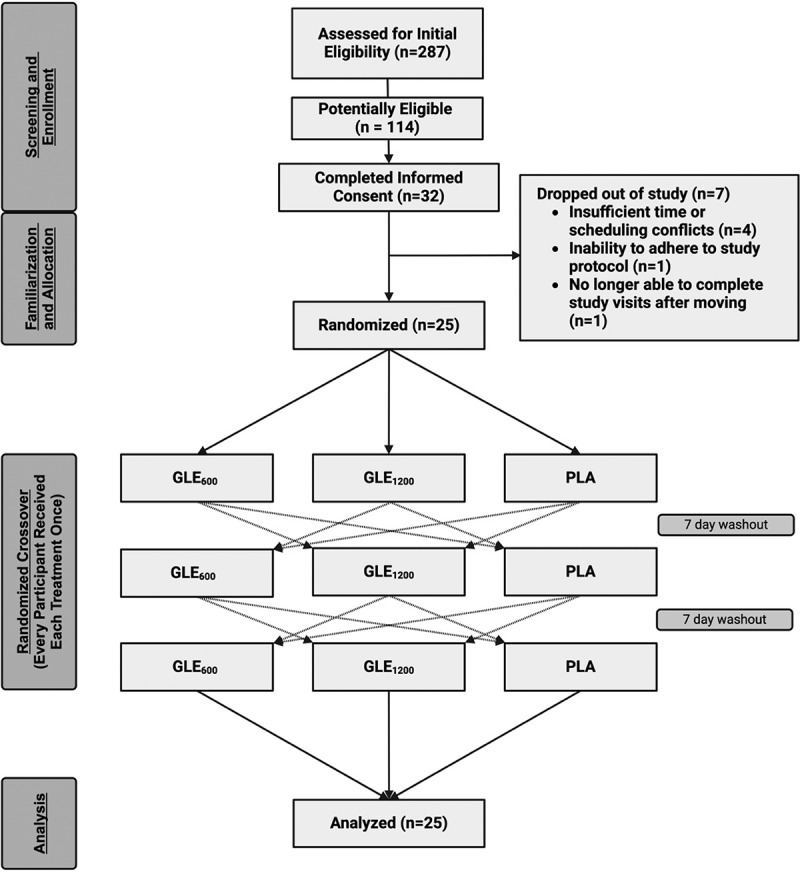
CONSORT flow diagram depicting the flow of participants through the study from initial screening to data analysis. Figure created with BioRender.com.

Two-hundred and eighty-seven individuals reviewed an informed consent summary through REDcap and completed initial screening for this study. Of these potential participants, 114 were deemed potentially eligible, and 32 filled out the informed consent and agreed to participate. Of the subjects who agreed to participate in the study, seven did not complete the study (Figure 1). The reasons provided for not completing the study included: an injury during activities unrelated to the study (*n* = 1), insufficient time or scheduling conflicts (*n* = 4), an inability to adhere to the study protocol (*n* = 1), and unable to complete study visits after moving away (*n* = 1).

Table 1 includes the characteristics for the 25 participants who enrolled in and completed this study. Participants were included in this study if they were between the ages of 18–45 y, physically active (≥150 min/wk or ≥75 min/wk of moderate or vigorous physical activity, or an equal combination) for at least 3 months, and otherwise healthy with a BMI <30 kg/m^2^. Participants were excluded if: they had a current injury that preluded exercise participation; or were currently utilizing nicotine, cannabis, prescription stimulants, ADD/ADHD, anti-depressant, or other central acting medication(s); or if they had previously been diagnosed with ADD/ADHD, clinical depression, or generalized anxiety disorder; or if they had a history of metabolic, hepatorenal, musculoskeletal, autoimmune, or neurologic disease; or if they were currently taking thyroid, hormonal, hyperlipidemic, hypoglycemic, anti-hypertensive, anti-inflammatory, or anti-coagulant medications; or if they were or had been treated for Metabolic Syndrome or had been clinically diagnosed with or were taking medication for a cardiometabolic-disorder (e.g. pre-diabetes, type II diabetes, high blood pressure, obesity, hypercholesterolemia, etc.); or if they were currently pregnant or lactating; or if they had been diagnosed with an allergy to any ingredient present within the study treatments; or if they were a current competitive NCAA athlete; or if they indicated or demonstrated an unwillingness to comply with the controls and conditions of the study. Prior to all testing, participants were instructed to restrict caffeine usage to less than 100 mg/day for at least 7 days prior to the first experimental visit and to continue restriction throughout the study. This study was approved by and carried out in accordance with the University’s Institutional Review Board for the protection of human subjects (IRB # 202202283; Approval Date: 05/16/2022) and was pre-registered (ClinicalTrials.gov ID NCT05928195).

**Table 1.** Participant demographic characteristics (*n* = 25).

Characteristic	Mean or n	SD or %
Sex		
Male	9	36%
Female	16	64%
Age (y)	28.3	6.7
Height (cm)	168.7	9.4
Weight (kg)	69.5	11.8
BMI (m/kg^2^)	24.4	3.4
Systolic BP (mmHg)	113	8
Diastolic BP (mmHg)	72	7
Habitual Caffeine Consumption (mg/day)	97	100

### 2.2. Experimental design

This study utilized a randomized, double-blind, placebo-controlled crossover design involving a familiarization visit and then three experimental visits separated by 7 days. Following the 7-day washout period and familiarization to both cognitive and motor cognitive tests, the participants arrived for experimental visits in a fasted state and having abstained from alcohol, caffeine, and strenuous exercise for the previous 48 h. Upon arrival, participants also provided a dietary food log in which they recorded all dietary intake for the preceding 24 h. Participants then laid supine on a padded medical examination table and rested quietly for 10 min. Following the initial 10-min rest period, blood pressure and heart rate were measured, followed by a 12-lead ECG assessment. The participant then consumed the supplement and proceeded to sit quietly and rest in a separate room. In order to reduce cognitive stimulation during this period, use of phones or computers was prohibited, as was reading and writing. Instead, participants were given picture books containing scenic landscape photography to prevent severe boredom and to standardize the stimulus during the waiting period [5]. Forty-minutes after supplementation, blood pressure, heart rate, and ECG assessments were repeated. One-hour after GLE or PLA consumption, participants completed a validated, reliable battery of computer-based cognitive tests (CNS Vital Signs CNSVS, v. 4.0.95, Morrisville, North Carolina), which was immediately followed by completion of the Short Form of The Profile of Mood States questionnaire (POMS-SF) and visual analog scales assessing supplement-related jitteriness and focus [17]. Finally, participants completed four different motor-cognitive (neurocognitive hop) tests: the single-leg central-reaction, single-leg memory, single-leg peripheral-reaction crossover test, and single-leg pursuit tests [18]. All experimental visits were identical in conduct and were overseen by the same investigators. Following completion of the last experimental visit, participants completed a condition blinding questionnaire where they indicated at which experimental visit they believed they consumed each of the drinks.

### 2.3. Familiarization

Based on the initial eligibility determination, participants were invited to the laboratory to complete the informed consent process. Immediately following consent, height and weight were measured, and participants with a confirmed BMI <30 m/kg^2^ were invited to complete additional questionnaires to obtain information regarding their demographics, health history, and habitual caffeine use to verify final eligibility. Participants were then familiarized to the cardiovascular, cognitive, and motor-cognitive hop assessments.

### 2.4. Lifestyle controls

Participants tracked their nutrition, caffeine use, and supplementation the day prior to the experimental visit. These data were entered into ESHA Food and Nutrition Database (ESHA Research, Oak Brook, IL) which provided calculations of nutritional intake including total energy (kcals/day), protein (g/day), fat (g/day), carbohydrates (g/day) and caffeine (mg/day). Habitual caffeine consumption was also assessed prior to the experimental visits (Caffeine Consumption Questionnaire, 2010).

### 2.5. Supplementation

The participants received either placebo or organic guayusa extract (AmaTea® Max, Applied Food Sciences) in one of two doses − 600 mg (120 mg caffeine) or 1200 mg (240 mg caffeine) – in randomized order during the 3 experimental visits. The randomization scheme was produced in blocks of 6 and counterbalanced using an online randomization generator (www.randomization.com) by an investigator who oversaw administration but was not directly involved in data collection (NDMJ). In each condition, participants consumed two vegan capsules that were matched for packaging and appearance, where both capsules contained AmaTea® in the 1200 mg condition (GLE_1200_), 1 capsule contained AmaTea® and 1 contained maltodextrin in the 600 mg condition (GLE_600_), and both capsules contained maltodextrin in the placebo condition. Capsules were taken with an 8 oz glass of water. The placebo was devoid of any other active ingredients. The active compounds in AmaTea® Max included: caffeine (20 wt/wt%; 120 mg and 240 mg in GLE_600_ and GLE_1200_, respectively), chlorogenic acids (30 wt/wt%; 180 mg and 360 mg in GLE_600_ and GLE_1200_, respectively), and other polyphenols, including quercetin glucosides (2–5%), kaempferols (1–3%) and the secondary chlorogenic acid esters, caffeoylquinic acids, p-coumaroylquinic acids, and feruloylquinic acids (8–12%). Supplements were manufactured, packaged, and provided by Applied Food Sciences (Kerrville, TX, USA), who blinded and labeled the supplement as “A,” “B,” or “C.” Prior to blinding and labeling, each lot’s contents were also validated at a second facility (BVC Innovation Lab, 2500 Crosspark Road, Coralville, IA; 2/21/2022), which confirmed that each capsule contained 20.3 wt/wt% caffeine, 30.5 wt/wt% chlorogenic acids, and met the microbe requirements of the United States Pharmacopoeia, and solubility was 99.79%. Researchers remained blinded until the completion of all analyses. Blinding was broken by NDMJ following collection of all data and after performing all primary data analyses.

### 2.6. Cognitive performance

Cognitive performance was assessed using CNS Vital Signs (CNSVS, v. 4.0.95, Morrisville, North Carolina), a validated, reliable computerized neurocognitive test battery for use in clinical and research settings. The battery was adapted from 10 normed neurocognitive tests (Carpenter et al. 1990; Gualtieri and Johnson 2006; Riccio et al. 2002) and produced assessment scores across 15 clinical domains of neurocognitive function. The neurocognitive tests used included the Verbal Memory Test, Visual Memory Test, Finger Tapping Test, Symbol Digit Coding, Stroop Test, Shifting Attention Test, Continuous Performance Test, Perception of Emotions Test, Non-Verbal Reasoning Test, and the Four Parts Continuous Performance Test. The clinical domains of neurocognitive function measured from these tests were as follows: composite memory, verbal memory, visual memory, psychomotor speed, reaction time, complex attention, cognitive flexibility, processing speed, executive function, simple attention, motor speed, social acuity, reasoning, sustained attention, and working memory. For all domains except for complex attention and reaction time, higher scores indicate greater cognitive performance. For complex attention and reaction time, lower scores represent greater cognitive performance.

### 2.7. Profile of mood states

Moods were assessed using the Profile of Mood States (POMS, 2nd Edition, Multi-Health Systems, North Tonawanda, NY), a commonly used and validated [27–29] psychological screening instrument to examine transient affective states comprising 35 items (adjectives). At the end of the cognitive tests, participants indicated the extent to which they felt each item during the elapsed time since consuming the supplement on a 5-point scale ranging from “not at all” (scored 0) to “extremely” (scored 4). Seven mood states were assessed: Anger-Hostility, Confusion-Bewilderment, Depression-Dejection, Fatigue-Inertia, Tension-Anxiety, Vigor-Activity, and Friendliness. Total Mood Disturbance, an indication of global psychological distress and the primary POMS-derived outcome in this study, was derived as the sum of Anger-Hostility, Confusion-Bewilderment, Depression-Dejection, Fatigue-Inertia, Tension-Anxiety, and Vigor-Activity, with Vigor-Activity weighted negatively [27].

### 2.8. Perceived energy, focus, and jitteriness

Subjective feelings were assessed utilizing a visual analog scale, in which a score of zero signified the lowest rating (indicating no feeling at all or being at the absolute lowest value on the scale) and a score of 100 represented the highest rating (indicating an extreme feeling or being at the absolute highest value on the scale) [11,30,31]. The participants recorded their ratings for energy, focus, and jitteriness immediately following completion of the POMS.

### 2.9. Motor-cognitive testing

Participants completed four different motor-cognitive (neurocognitive hop) tests: the single-leg central-reaction, single-leg memory, single-leg peripheral-reaction crossover, and single-leg pursuit tests. In these tests, a light system flashed random colors and participants stood and hopped on one leg for all tests. Testing was performed on both legs, and scores for each test were averaged across leg. For the single-leg central reaction test, when the correct color (e.g. green) flashed, participants hopped as fast and as far as possible with a single-hop. For the single-leg memory triple hop, one of six colors was randomly selected at the start of each trial and when that color flashed, the participant was instructed to hop forward as fast and as far as possible three times (i.e. 3 consecutive hops) without falling or touching the opposite foot to the ground. For the single-leg peripheral-reaction crossover test, two lights were placed in each side of the participant’s peripheral vision, and both lights were capable of flashing either red or green. Once the color flashed green, the participants executed a series of hops depending on whether the left or right light flashed. For example, if the right light flashed green, the participants hopped right, left, and then right forward over a piece of tape on the floor without falling or placing the other foot down. For the single-leg pursuit test, lights were placed 6-m away and directly in front, 45° to the left, and 45° to the right of the participants. At a random time, one light blinked either red (don’t hop) or green (hop). Participants waited until a green light flashed and then hopped to the respective light as fast as possible.

### 2.10. Cardiovascular assessments

Cardiovascular outcomes included blood pressure, heart rate, oxygen saturation and cardiac electrical activity via electrocardiogram (ECG), which were measured at baseline and ~55 min after supplement consumption and immediately prior to completion of the cognitive testing battery. Blood pressure was measured using an automatic, upper-arm, electronic sphygmomanometer (Omron Platinum BP5450, Omron Healthcare, Lake Forest, IL) placed on the right arm 2 cm from the antecubital space. All blood pressure readings were taken with the participant lying supine at a 45° angle in a relaxed and supported position. Two blood pressure measurements were obtained during each assessment. If the variance in either systolic or diastolic blood pressures exceeded 5 mmHg, a third reading was obtained and the closest two recordings were averaged and used for analyses. Heart rate and oxygen saturation (Sp02) were measured using a fingertip pulse-oximeter (Innovo iP900AP, Innovo Medical, Stafford, TX) placed on the left index finger. Average oxygen saturation (%) and heart rate (BPM) was determined by calculating the mean of two readings.

Cardiac electrical activity was assessed with a 12-lead electrocardiogram (ECG) (CardioTech SE-12 Series, Edan Instruments, Pingshan District, P.R. China). A 30-s continuous ECG was obtained with participants lying supine at a 45° angle, while relaxed and fully supported. The primary outcome from each ECG recording was heart-rate corrected QT interval (QTc, ms), which is an indirect measure of ventricular repolarization that is associated with incidence of cardiac arrhythmias and sudden death [23,24].

### 2.11. Statistical analyses

#### 2.11.1. A priori sample size estimation

Sample size was determined a priori using the planned analyses (one-way repeated measures analysis of variance (ANOVA) with one group and three measurements) with an estimated effect size of f = 0.25, 1-β set to 0.9, and the correlation between repeated measurements set at 0.7. The sample size necessary to observe this effect was 22 subjects, so we planned recruitment of at least 28 participants to conservatively finish with a total of at least 24 subjects.

#### 2.11.2. Statistical analysis

Descriptive statistics were calculated to summarize participant demographic characteristics. Normality of residuals were inspected using Q–Q plots, and log-transformation was performed as necessary. Consistent with our a priori defined analysis plan, one-way (GLE_600_ vs. GLE_1200_ vs. PLA) ANOVAs (with Greenhouse–Geisser correction where epsilon < 0.75) were used to examine condition effects on cognitive and motor-cognitive performance, mood, energy, focus, and jitteriness. Two-way repeated measures ANOVAs were used to examine the interaction of condition (GLE_600_ vs. GLE_1200_ vs. PLA) with time (pre- vs. post-supplementation) for resting cardiovascular outcomes. Two-way (condition × sex) repeated measures analyses of variance (ANOVAs) were also conducted to examine whether condition effects were dependent on biological sex and were reported significant. When appropriate, follow-up analyses included lower order repeated measures ANOVAs and/or Ηolm-Sidák-corrected dependent, multiple-comparison tests. Mean ± standard error of differences are provided for post-hoc pairwise comparisons where appropriate. All statistical analyses were performed using GraphPad Prism (v.9.2.0, San Diego, CA, USA) and the a priori alpha was set at 0.05.

## 3. Results

### 3.1. Lifestyle controls

There were no significant between-treatment differences in nutritional intake measures, which included kcals (*p* = 0.74), protein (*p* = 0.67), carbohydrates (*p* = 0.50), and fat (*p* = 0.99) during the week preceding each experimental visit.

### 3.2. Caffeine dosing

We chose to utilize absolute caffeine dosing in this study to maximize ecological validity, as most purported nootropic, caffeine-containing supplements and/or energy drinks contain a fixed absolute dose. In this study, GLE_600_ and GLE_1200_ provided 1.54 ± 0.21 and 3.08 ± 043 mg/kg of caffeine in men and 1.90 ± 0.24 and 3.81 ± 0.49 mg/kg of caffeine in women. Thus, the relative caffeine doses provided by GLE_600_ (+0.36 ± 0.15 mg/kg; *p* = 0.02) and GLE_1200_ (+0.72 ± 0.15; *p* < 0.001) were greater in women than men.

### 3.3. Mood states

Significant treatment effects were observed for total mood disturbance (F_2,48_ = 3.47; *p* = 0.039) and fatigue-inertia (F_2,48_ = 5.09; *p* < 0.01). Acute GLE_600_ consumption reduced total mood disturbance (−6.88 ± 2.61 a.u. [−187%]; *p* = 0.034) and fatigue-inertia (−2.84 ± 0.89 a.u. [−65.1%]; *p* = 0.008) when compared to placebo, while GLE_1200_ had no significant effect on either (*p* = 0.30 and 0.14, respectively). The treatment effect was not significant for vigor-activity, confusion-bewilderment, or depression-dejection (all F_2,48_ = 2.50–2.89, *p* = 0.065–0.093), nor for tension-anxiety, anger-hostility, or friendliness (all F_2,48_ ≤1.13, *p* = 0.33–0.54).

### 3.4. Perceived energy, focus, and jitteriness

**Figure 2.**
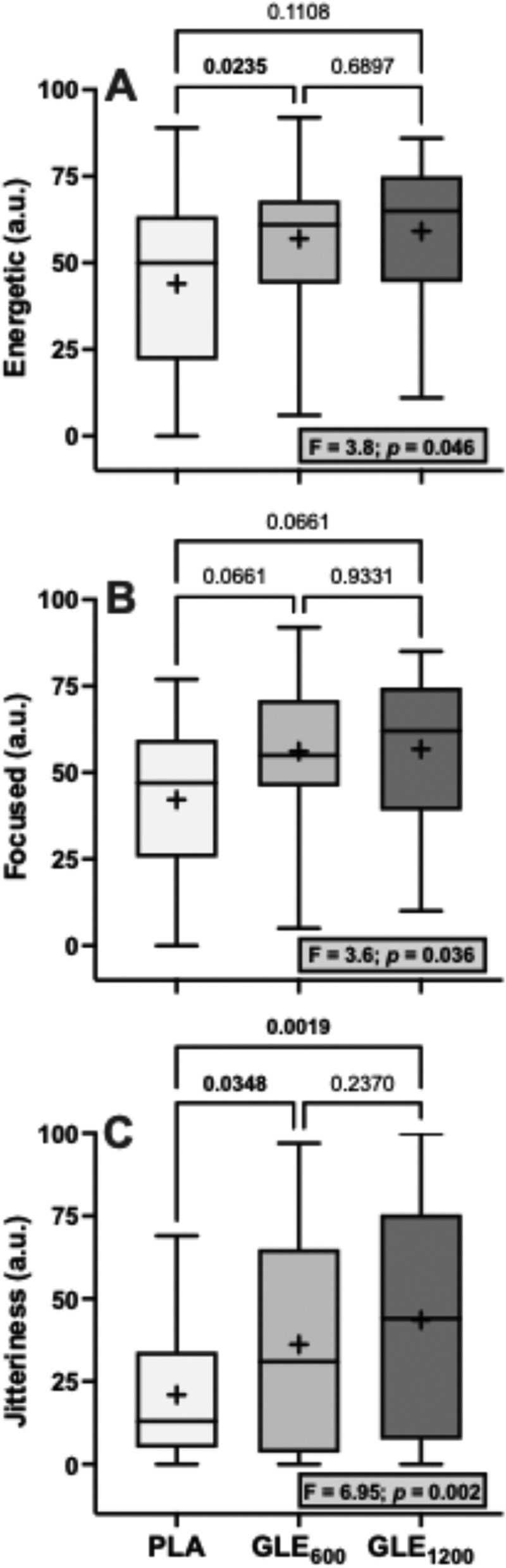
The effect of treatment (GLE_600_ vs. GLE_1200_ vs. PLA) on perceived energy (panel A), focus (panel B), and jitteriness (panel C) (all *n* = 25). The effect of treatment determined by one-way ANOVAs are shown in inset text boxes. P-values from Holm-Sidak’s multiple post-hoc comparisons are shown above brackets between treatments.

**Figure 3.**
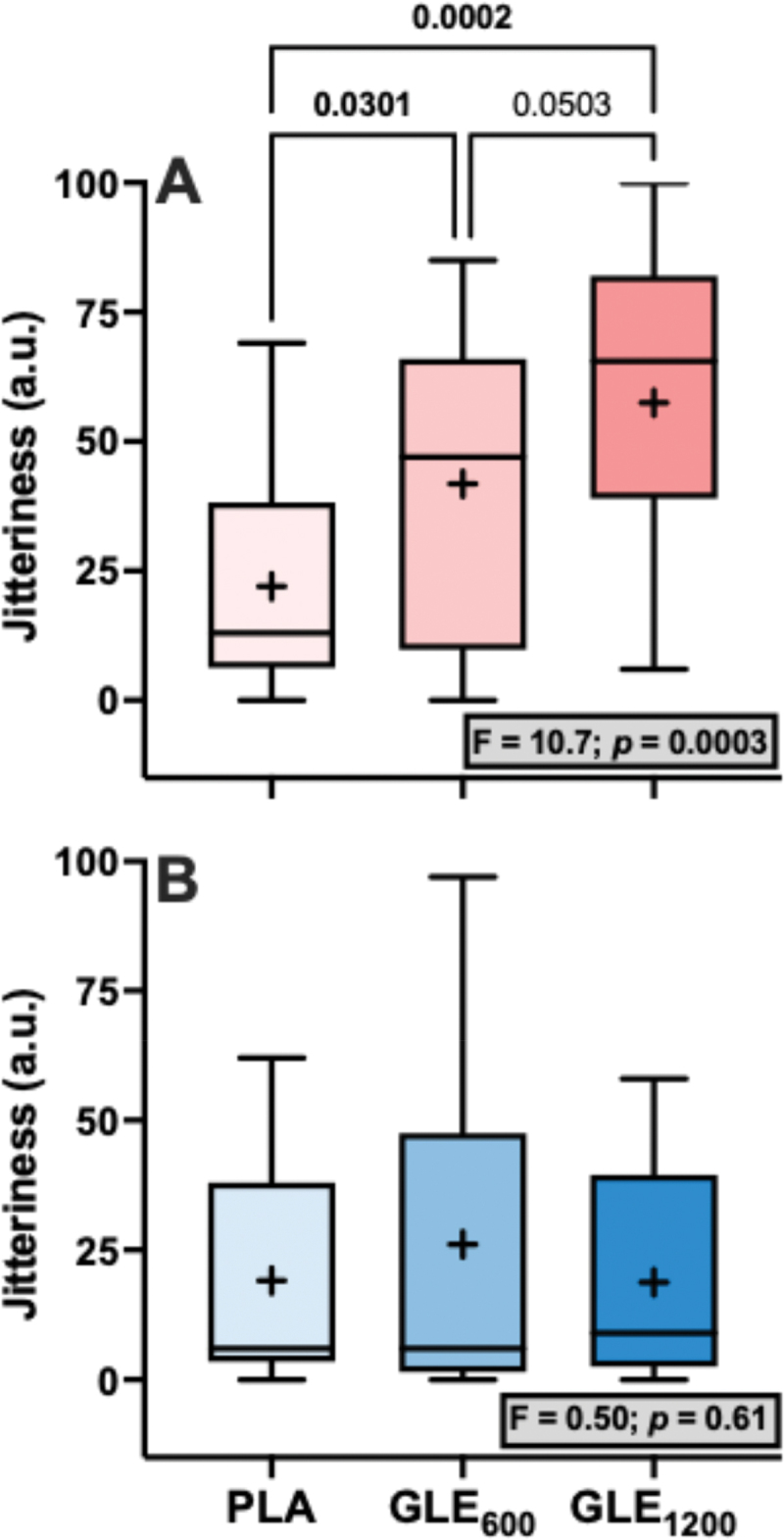
The effect of treatment (GLE_600_ vs. GLE_1200_ vs. PLA) on perceived jitteriness in female (panel A, *n* = 16) and male (panel B, *n* = 9) adults. The effect of treatment determined by one-way ANOVAs are shown in inset text boxes. P-values from Holm-Sidak’s multiple post-hoc comparisons are shown above brackets between treatments.

Significant treatment effects for energy (F_1.4,34.1_ = 3.80, *p* = 0.046), focus (F_2,48_ = 3.58, *p* = 0.036), and jitteriness (F_2,48_ = 6.95, *p* = 0.002) were observed (Figure 2). Perceived energy was significantly greater in GLE_600_ (+13.00 ± 4.49 a.u. [+29.5%]; *p* = 0.02) but not GLE_1200_ (+15.16 ± 7.58 a.u [+34.5%]; *p* = 0.11) when compared to PLA. Perceived focus tended to be but was not significantly greater in GLE_600_ (+14.00 ± 4.91 a.u. [+33.0%]; *p* = 0.066) or GLE_1200_ (+14.52 ± 6.87 a.u. [+34.4%]; *p* = 0.066) than in PLA. Lastly, perceived jitteriness was greater in both GLE_600_ (+15.20 ± 4.63 a.u. [+72.5%]; *p* = 0.035) and GLE_1200_ (+22.60 ± 7.22 a.u [+107.8%]; *p* = 0.002) compared to PLA. However, the effect of GLE dose on jitteriness was sex-dependent (condition × sex: F_2,46_ = 4.57, *p* = 0.015; Figure 3). Jitteriness did not change in men (F_2,16_ = 0.50; *p* = 0.61). However, jitteriness increased (F_2,30_ = 10.7; *p* = 0.003) in a dose-dependent manner in women such that jitteriness was greater in GLE_600_ (+19.8 ± 7.7 a.u. [+90.0%]; *p* = 0.03) and GLE_1200_ (+35.5 ± 7.7 a.u. [+161.4%]; *p* < 0.001) than in PLA, and in GLE_1200_ than in GLE_600_ (+15.7 ± 7.7 a.u. [+37.5%]; *p* = 0.050).

### 3.5. Cognitive performance

**Figure 4.**
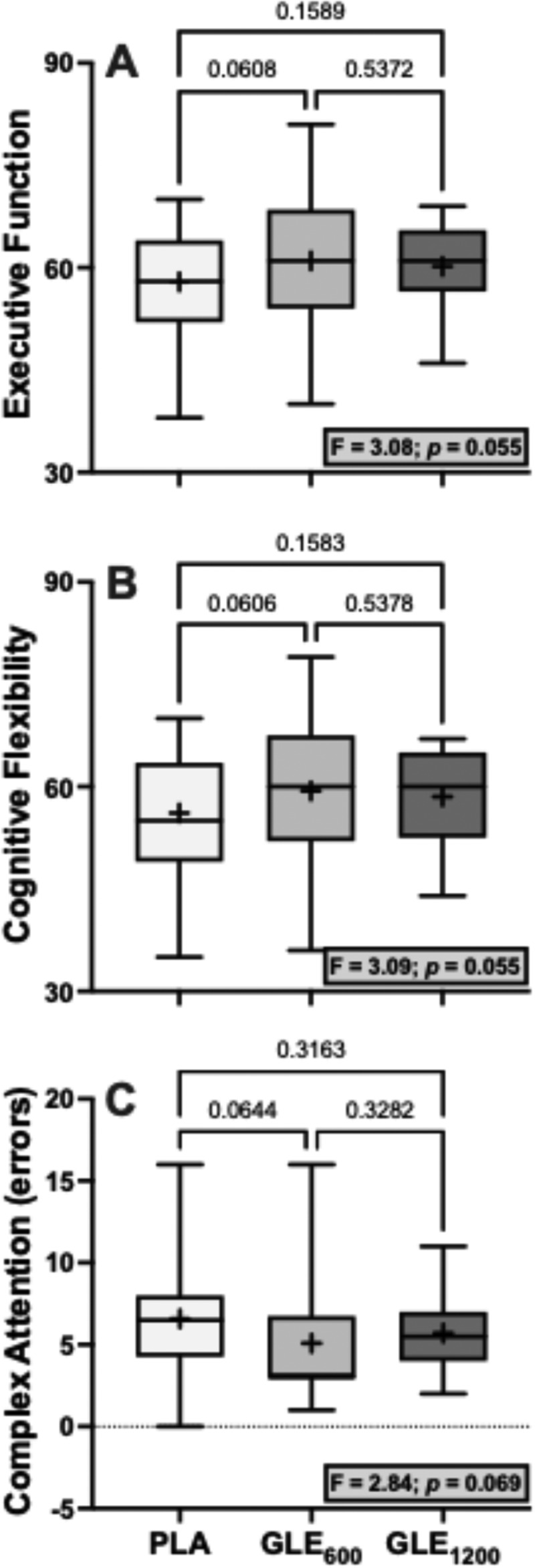
The effect of treatment (GLE_600_ vs. GLE_1200_ vs. PLA) on executive function (panel A, *n* = 25), cognitive flexibility (panel B, *n* = 25), and complex attention (panel C, *n* = 24). The effect of treatment determined by one-way ANOVAs are shown in inset text boxes. P-values from Holm-Sidak’s multiple post-hoc comparisons are shown above brackets between treatments.

There were significant treatment effects for motor (F_2,48_ = 5.29; *p* = 0.008) and psychomotor speed (F_2,48_ = 5.89; *p* = 0.005). Acute GLE_600_ consumption significantly improved motor (+4.52 ± 1.42 a.u. [+4.4%]; *p* = 0.008) and psychomotor speed (+7.20 ± 2.16 a.u. [+4.2%]; *p* = 0.005) when compared to placebo. GLE_1200_ consumption significantly improved psychomotor speed (+5.08 ± 2.16 a.u. [+2.9%]; *p* = 0.045), but did not significantly improve motor speed (+3.08 ± 1.42 a.u. [+3.0%]; *p* = 0.069) when compared to placebo. Treatment effects for executive function (F_2,48_ = 3.08; *p* = 0.055), cognitive flexibility (F_2,48_ = 3.09; *p* = 0.055), and complex attention (F_2,47_ = 2.84; *p* = 0.069) were not statistically significant (Figure 4). There were also no significant treatment effects observed for composite memory, working memory, processing speed, reasoning, sustained attention, or simple attention when comparing PLA to both GLE_600_ and GLE_1200_ (all *p* ≥0.19).

### 3.6. Motor cognitive performance

**Figure 5.**
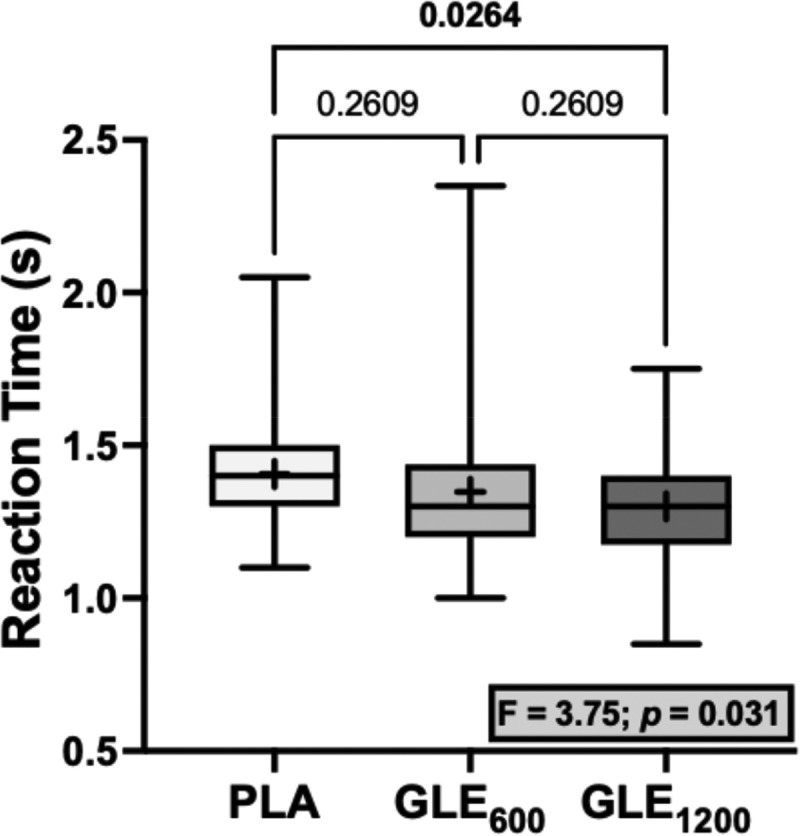
The effect of treatment (GLE_600_ vs. GLE_1200_ vs. PLA) on motor-cognitive reaction time during the single-leg peripheral crossover test (*n* = 25). The effect of treatment determined by a one-way ANOVA is shown in the inset text box. P-values from Holm-Sidak’s multiple post-hoc comparisons are shown above brackets between treatments.

No significant differences were observed among treatments for jump distance for any of the tests (all F_2,48_ = 0.16–0.62, *p*’s ≥ 0.540). Similarly, no significant differences were observed among treatments for the time to completion (F_2,48_ = 0.44; *p* = 0.645) or the reaction time in the single-leg pursuit test (F_2,48_ = 2.56; *p* = 0.088). However, there was a significant treatment effect for reaction time in the single-leg peripheral crossover test. Reaction time was faster in the GLE_1200_ condition than the placebo (+1.300 ± 0.442 vs. +1.406 ± 0.038 ms; *p* = 0.017; Figure 5).

### 3.7. Cardiovascular outcomes

There was a significant treatment × time interaction for SBP (F_1.8,41.4_ = 6.18, *p* = 0.0058). Relative to PLA, SBP increased in GLE_600_ (+4.5 ± 1.3 mmHG; *p* = 0.004) and GLE_1200_ (+3.9 ± 1.6 mmHg; *p* = 0.022). There was also a treatment × time interaction for DBP (F_1.98,45.5_ = 7.68, *p* = 0.0014). Relative to PLA, DBP increased in GLE_600_ (+4.4 ± 1.4 mmHG; *p* = 0.0045) and GLE_1200_ (+5.2 ± 1.3 mmHg; *p* = 0.0015).

There was no significant treatment × time interaction for HR (F_1.8,43.2_ = 0.83; *p* = 0.43), but there was a significant main effect of time (F_1,24_ = 42.94, *p* < 0.0001). When collapsed across condition, HR increased from pre- to post-supplementation (+5.8 ± 0.9 bpm; *p* < 0.0001). There was also no significant treatment × time interaction for QTc (F_1.85,44.3_ = 1.19, *p* = 0.31), but there was a main effect for time (F_1,24_ = 4.64, *p* = 0.042). When collapsed across treatment, the QTc decreased from pre- to post-supplementation (−4.6 ± 2.1 ms, *p* = 0.042).

## 4. Discussion

This is the first study to examine the dose-dependent effects of acute GLE supplementation on mood, perceived energy, focus, jitteriness, cognitive and motor cognitive performance, and on heart rate, blood pressure, and ventricular repolarization. Our findings indicate that acute GLE supplementation improves cognitive and motor-cognitive performance, mood, and perceived energy. Notably, these effects appear to be dose-dependent. Specifically, GLE_600_ significantly improved motor speed, psychomotor speed, total mood disturbance, fatigue-inertia, and perceived energy; GLE_1200_ also improved psychomotor speed but uniquely increased motor-cognitive performance as reflected by a decrease in reaction time during a neurocognitive hop test. Further, the effect of GLE on jitteriness was both dose- and sex-dependent, whereby jitteriness increased with increasing GLE dose in women only. Both GLE_600_ and GLE_1200_ similarly increased systolic and diastolic blood pressures by 4–5 mmHg. Finally, neither GLE_600_ nor GLE_1200_ acutely influenced QTc length. Therefore, the goal of supplementation should be considered when selecting a GLE dosing strategy, with GLE_600_ likely optimizing cognitive and mood-related outcomes while limiting side-effects such as jitteriness in women, and GLE_1200_ promoting the only notable improvement in motor-cognitive performance.

**Figure 6.**
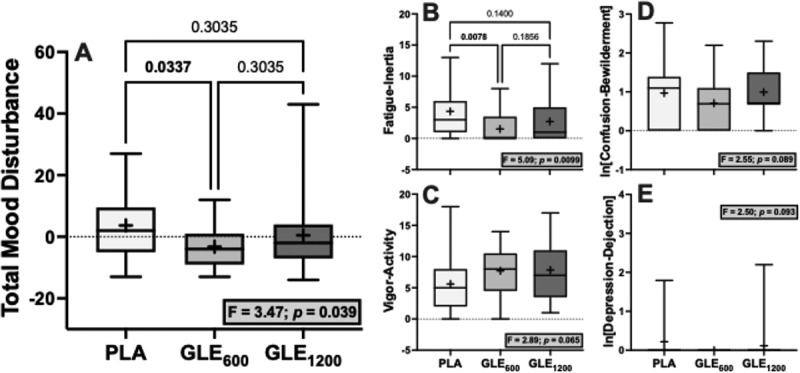
The effect of treatment (GLE_600_ vs. GLE_1200_ vs. PLA) on total mood disturbance (panel A), fatigue-intertia (panel B), vigor-activity (panel C), confusion-bewilderment (panel D), and depression-dejection (panel E) (all *n* = 25). The effect of treatment determined by one-way ANOVAs are shown in inset text boxes. P-values from Holm-Sidak’s multiple post-hoc comparisons are shown above brackets between treatments where appropriate.

In the only prior study to our knowledge to examine the effect of GLE on mood, Bloomer et al. [17] reported that a 1,080 mg dose of GLE had no effect on mood measured using the Brunel Mood Scale. However, in the present study GLE_600_ improved total mood disturbance (Figure 6a), in part by significantly decreasing fatigue-inertia (Figure 6b) and by non-significantly improving other mood-states (e.g. vigor-activity, confusion-bewilderment, Figure 6c-e). Notably, GLE_1200_ had no significant effect on mood when compared to placebo in the present study. Together with the findings of Bloomer et al. [17], these data suggest that the mood-enhancing benefits of GLE are optimized at low doses and that doses ≥1,080 mg do not provide any additional benefit and may even be counter-productive for mood enhancement. In support of this conclusion, prior evidence also appears to indicate that lower doses of caffeine (≤200 mg) seem to maximize improvements in mood in response to acute caffeine supplementation [32].

Bloomer et al. [17] also reported that a 1,080 mg GLE dose had no influence on perceived jitteriness, focus, or energy. In contrast, we observed that both GLE_600_ and GLE_1200_ increased jitteriness relative to placebo, and that this effect was strongest in GLE_1200_. Further, this effect was dependent on sex. In females jitteriness increased in a dose-dependent manner (Figure 3a), but did not in males (Figure 3b). Thus, one potential explanation for the lack of increase in jitteriness previously observed by Bloomer et al. [17] was that their sample was composed of 47 males to 2 females (e.g. 96% male) participants. This finding is also in alignment with prior evidence indicating that females are more likely to report negative side-effects such as anxiety, nervousness, palpitations, and headache and negative subjective responses than males in response to the same relative doses of caffeine, [33,34] and also that caffeine intake is associated with higher odds of generalized anxiety in females, but not males. [35] Therefore, it appears that the effect of GLE on perceived jitteriness is dependent on sex, and females who supplement with GLE may wish to avoid higher doses to avoid this side-effect. Finally, we also observed significant GLE-related improvements in energy. However, much like GLE’s effects on mood, only the GLE_600_ dose produced a significant improvement in perceived energy relative to placebo. Thus, again, the discrepancy observed between the study by Bloomer et al. [17] and ours may be explained by this apparent dose-dependent effect on mood and perceived energy.

**Figure 7.**
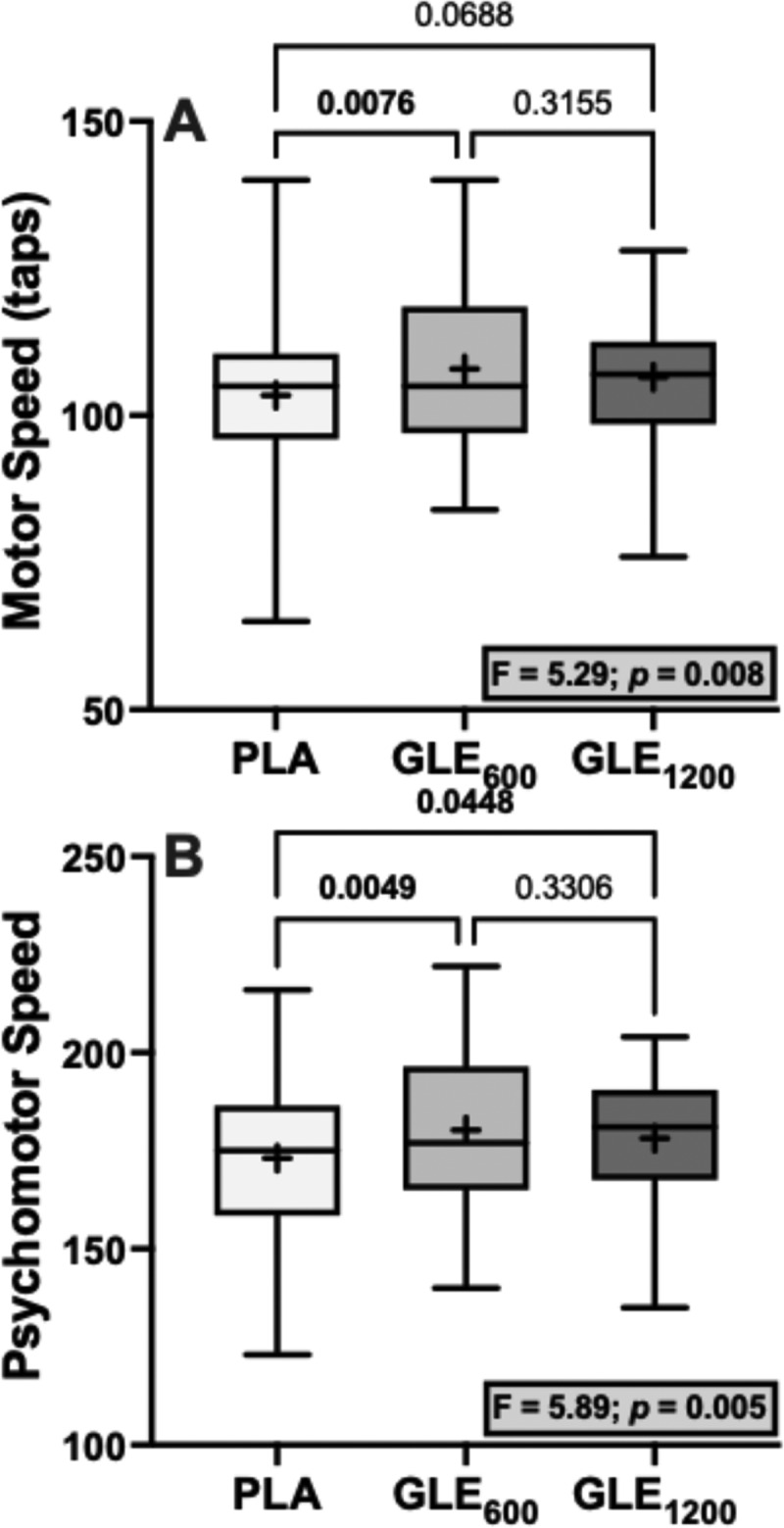
The effect of treatment (GLE_600_ vs. GLE_1200_ vs. PLA) on motor speed (panel A) and psychomotor speed (panel B) (all *n* = 25). The effect of treatment determined by one-way ANOVAs are shown in inset text boxes. P-values from Holm-Sidak’s multiple post-hoc comparisons are shown above brackets between treatments.

In the present study, GLE improved both motor and psychomotor speed, but similar to our findings for mood, this effect was strongest following GLE_600_ (Figure 7). We also observed non-significant treatment effects on executive function, cognitive flexibility, and complex attention (*p’s* = 0.055–0.069; Figure 4) driven by a tendency for GLE_600_ to improve each of these cognitive outcomes relative to placebo (*d’s =* 0.479–0.484). Thus, these findings indicate that low doses of GLE cause cognitive performance enhancements related to visual-perceptual responses and motor speed and coordination, and potentially to rapid decision-making and mental vigilance, adaptability, and flexibility. We have previously shown that a novel energy drink containing 200 mg of caffeine improves cognitive flexibility, executive function, motor speed, and psychomotor speed, among other cognitive domains, and that improvements in psychomotor speed were associated with improvements in a game that stresses visuospatial working memory (e.g. Tetris) [5]. Thus, it is plausible that the cognitive performance enhancements observed herein may translate to improved gaming performance. Although Bloomer et al. [17] reported that GLE did not influence performance in the video game “Fortnite,” the dose utilized in that study (1,080 mg) was more similar to the high-dose (1,200 mg) used herein, which was less effective. As previously described [36,37], there is an association between arousal and performance described by an inverted-U function, such that increasing arousal improves performance up to a point at which further increases in arousal will cause progressive decreases in performance. Further, it was not possible to control the skill of other players or the situations encountered from match to match in the video game deployed by Bloomer et al. [17], which may have introduced significant variability that made it difficult to detect a performance enhancing benefit. Thus, future studies may wish to reexamine the efficacy of GLE using a lower dose (e.g. GLE_600_) in a more well-controlled, sensitive video gaming environment [5].

**Figure 8.**
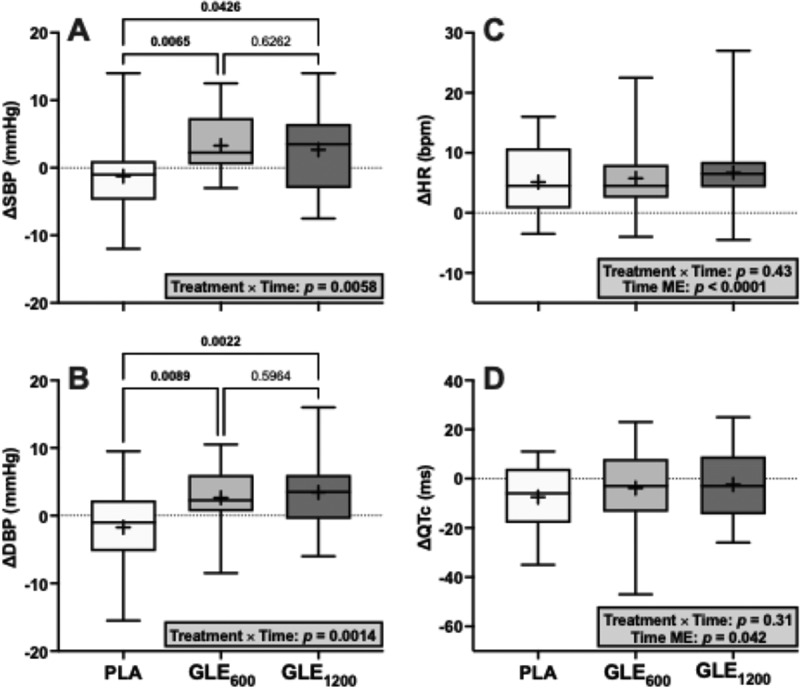
The effect of treatment (GLE_600_ vs. GLE_1200_ vs. PLA) and time (pre- vs. post-consumption) on systolic blood pressure (panel A), diastolic blood pressure (panel B), heart rate (panel C), and QTc interval (panel D) (all *n* = 25). Interaction and main effect (as appropriate) are shown in inset text boxes. P-values from Holm-Sidak’s multiple post-hoc comparisons are shown above brackets between treatments.

We also hypothesized that GLE would increase blood pressure in a dose-dependent manner, but minimally effect HR based on a prior meta-analysis suggesting that caffeinated drinks containing <200 mg versus ≥200 mg of caffeine elicit a 3.7 versus 6.4 mmHg increase in SBP without significantly influencing heart rate [26]. Partially consistent with this hypothesis, neither GLE_600_ nor GLE_1200_ influenced heart rate (Figure 8c). However, SBP increased similarly by 4.5 and 3.9 mmHg in response to GLE_600_ and GLE_1200_ relative to placebo (Figure 8a). Likewise, DBP increased similarly by 4.4 and 5.2 mmHg in response to GLE_600_ and GLE_1200_ relative to placebo (Figure 8b). Thus, although GLE did increase blood pressure, these increases were not dose-dependent and are generally consistent with those previously reported in response to caffeine-containing ingredients or energy drinks [5,26]. While these increases are relatively modest and not likely to be associated with increased acute cardiovascular risk in young, healthy populations, these elevations may be important for longterm risk if experienced consistently over the long term. Indeed, even 3–4 mmHg increases in blood pressure are associated with increased cardio- and cerebrovascular events at a population level [38]. Finally, as caffeine has been shown to produce stronger and more prolonged elevations in blood pressure in mildly hypertensive individuals, GLE-induced blood pressure elevations may be more dramatic and troublesome in those with chronic hypertension and/or when superimposed on other acute hypertensive stimuli (e.g. physical or mental stress) [39,40].

Prolongation of the QTc interval, which is an indirect measurement of ventricular repolarization, is an established risk factor for ventricular arrhythmias (e.g. torsades de pointes) [41]. A prolongation of the QTc interval that is ≥30 ms or an absolute QTc interval ≥500 ms is considered clinically significant and reason for careful follow-up [42]. Prior studies have indicated that energy drinks containing 300–320 mg of caffeine may produce worrisome increases in the QTc interval, with some individuals experiencing prolongation greater than 50 ms [20]. However, the greatest individual increases in QTc interval observed in the GLE_600_ and GLE_1200_ conditions in the present study were 23 ms and 25 ms, respectively. Furthermore, the greatest individual QTc interval observed at post-supplementation in the GLE_600_ and GLE_1200_ conditions was 457 ms in both conditions. Thus, not only were there no significant group changes in QTc interval in response to either GLE_600_ or GLE_1200_ in the present study, but there were also no individual responses observed that would be considered clinically noteworthy (Figure 8d). Therefore, our findings suggest that acute GLE supplementation at doses up to 1,200 mg is not likely to adversely influence ventricular repolarization.

## 5. Conclusion

Overall, our findings demonstrate that GLE_600_ is effective for improving motor and psychomotor speed, mood, and perceived energy, whereas GLE_1200_ was effective for enhancing psychomotor speed and reaction time in a neurocognitive hop test. Taken together, our findings point to an outcome-specific, dose-dependent effect of acute GLE supplementation that likely reflects the association between supplementation-related increases in arousal and cognitive versus physical performance. Accordingly, acute, lower-dose GLE supplementation optimized improvements in mood and cognitive performance in this study. On the other hand, GLE_1200_ did not generally cause improvements in mood or cognitive performance, but uniquely enhanced reaction time in a hop test. We also observed a dose-dependent increase in reported jitteriness, but this effect was sex-dependent and only apparent in females. Finally, GLE_600_ and GLE_1200_ similarly increased blood pressure, but did not significantly effect heart rate or ventricular repolarization on a group level. Our findings have important implications for GLE dosing, which should be dependent on the goal of supplementation and the target population.
